# Lyme-Associated Pericarditis: A Case Report and Literature Review

**DOI:** 10.7759/cureus.54096

**Published:** 2024-02-12

**Authors:** Muhammad Atif Ameer, Sridhar Reddy Patlolla, Nimi Patel, Rahul Mehta, Maham Babar

**Affiliations:** 1 Department of Medicine, Punjab Rangers Teaching Hospital, Lahore, PAK; 2 Hospital Medicine, Mission Hospital, Asheville, USA; 3 Internal Medicine, Suburban Community Hospital, East Norriton, USA; 4 Neurological Surgery, Philadelphia College of Osteopathic Medicine, Philadelphia, USA; 5 Department of Medicine, Khyber Medical University, Peshawar, PAK

**Keywords:** lyme disease, acute myopericarditis, st elevations, myocarditis complication, lyme's disease

## Abstract

Lyme disease is caused by *Borrelia burgdorferi (B. burgdorferi), which* is a spirochete transmitted by ticks of the genus *Ixodes*. Complications related to the cardiovascular system usually occur in the early phase of infection, and the most common cardiovascular complication of Lyme disease is atrioventricular block, especially third-degree heart block. We report a case of a young Caucasian male patient who presented to the emergency department (ED) with complaints of chest pain and shortness of breath. Initial investigations, including chest X-ray, were negative. An EKG revealed ST elevation and PR depression with troponin elevation. The echocardiogram showed a normal ejection fraction with no pericardial effusion. Skin examination was positive for erythema migrans concerning Lyme. Initial Lyme testing was negative in the patient and it should be repeated after four to six weeks, according to the guidelines. This case report highlights the importance of keeping the differentials broad in these patients even if the initial testing is negative, especially since misdiagnosis or delayed diagnosis can cause cardiac complications.

## Introduction

Lyme disease can involve multiple organ systems, and it is characterized by early cutaneous manifestations followed by a hematogenous spread with resultant cardiac, neurological, and musculoskeletal involvement. Lyme disease is treatable with antimicrobial drugs, and most patients recover fully, especially those who receive early and appropriate treatment [1]. It has been declared a “nationally notifiable condition" in the United States since 1991. Lyme is known to be the most prevalent tick-borne infection in the United States [2]. We present a case of a young Caucasian male from Pennsylvania with ST elevation and PR depression on EKG, along with troponin elevation, and erythema migrans.

## Case presentation

An 18-year-old male with a past medical history of asthma, irritable bowel syndrome, and obsessive-compulsive disorder (OCD) presented to the emergency department (ED) with complaints of chest pain. He reported that the chest pain had started in the morning at around 04:00 AM. The patient had attempted to relax by taking deep breaths without any improvement. His pain was 8/10 in intensity, located in the central chest, sharp, and had woken him up from sleep. The pain worsened with lying down, while taking deep breaths, and coughing. Leaning forward, however, reduced the pain. It was associated with shortness of breath and palpitations.

On further questioning, the patient reported a rash on his back that he had noticed a week ago while showering. According to the patient, the rash had progressively expanded in size over the last week, but it was not associated with any pain, itching, or burning sensation. The patient was unsure about receiving animal or tick bites and denied engaging in any outdoor activities. However, he resided in a deeply wooded area of eastern Pennsylvania. The patient denied fever, cough, chills, fatigue, arthralgia, nausea, dizziness, leg swelling, or diaphoresis.

**Figure 1:**
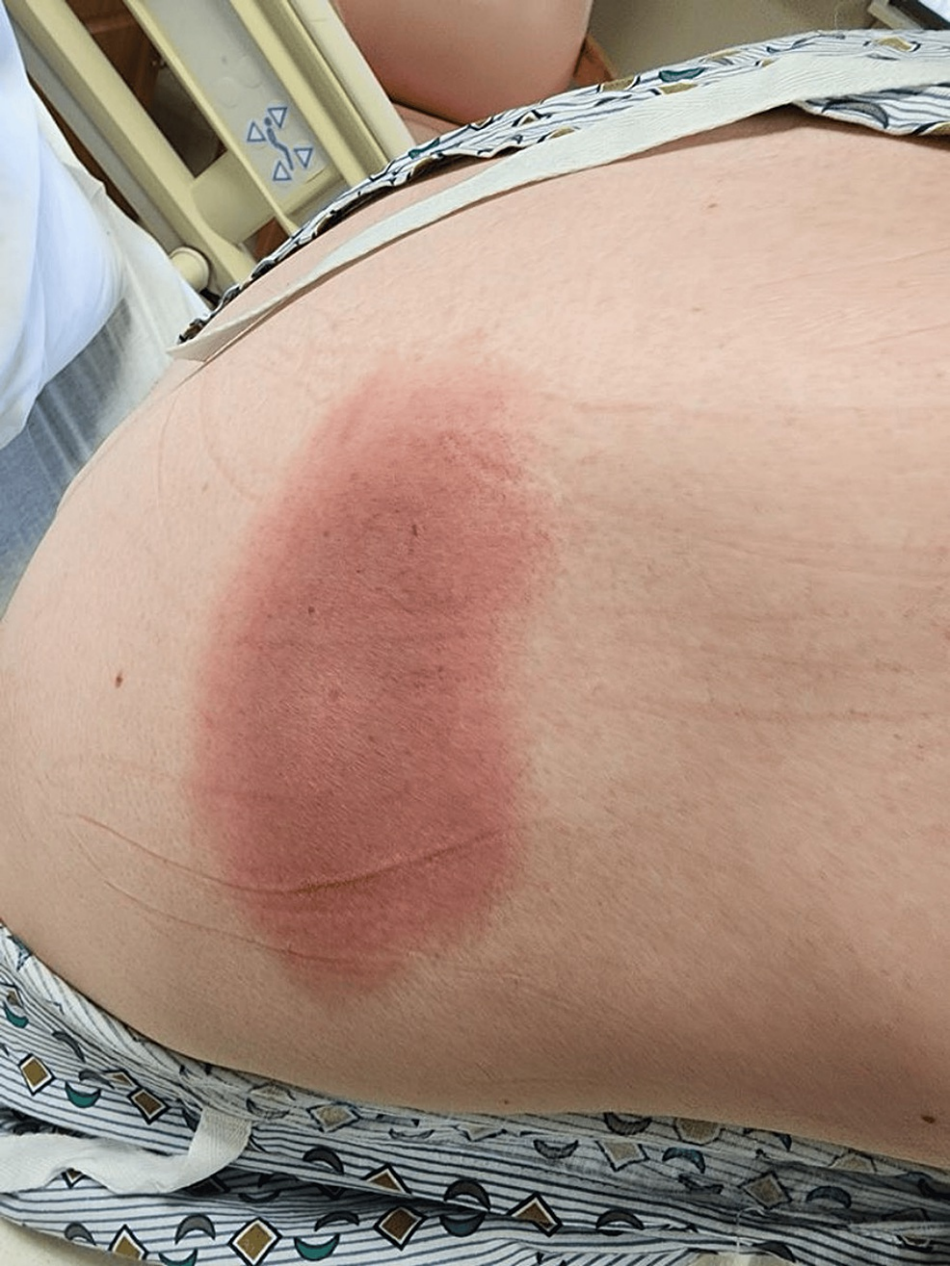
Right scapular Lyme rash with evolving central clearing on the day of admission (target lesion)

**Figure 2:**
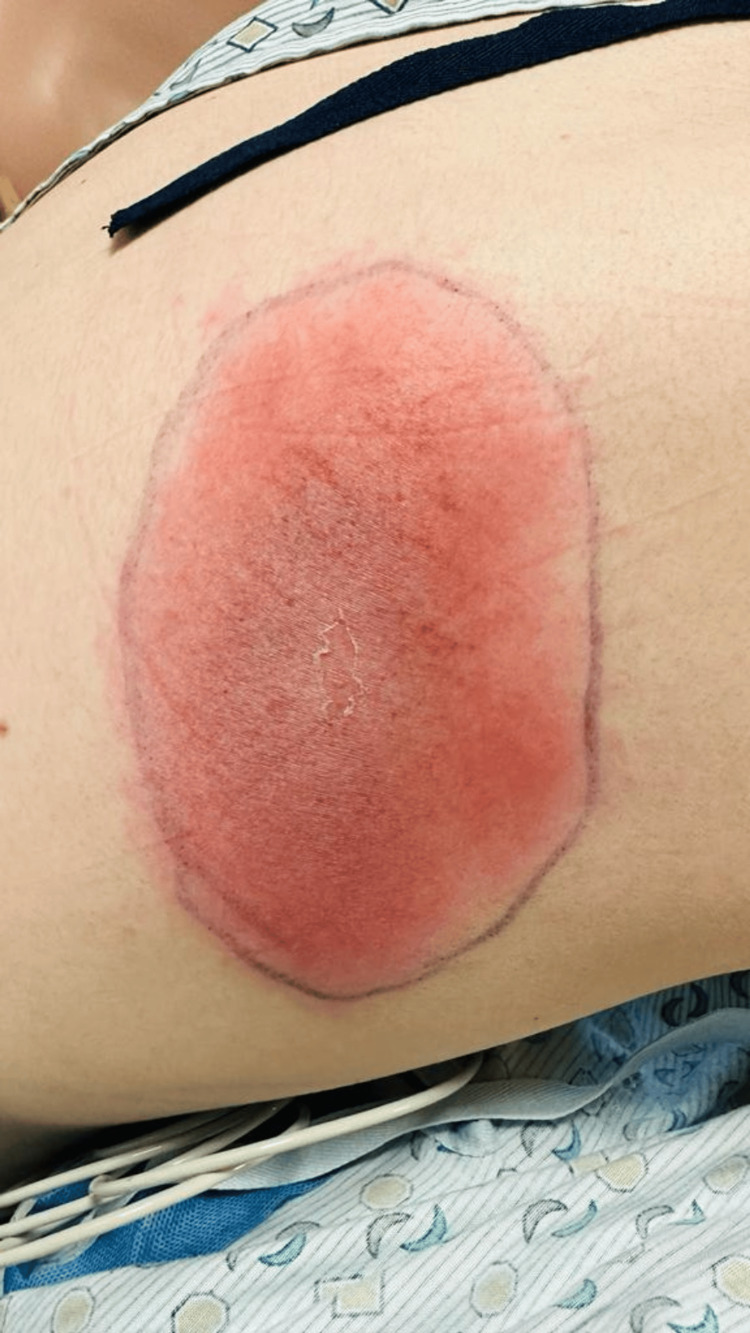
Right scapular Lyme rash with evolving central clearing (day 3)

During triage, the vitals were as follows - temperature: 96 °F, heart rate: 91/minute, respiratory rate 16/minute, blood pressure: 125/80 mmHg, and the patient was saturating 100% on room air. Physical examination showed an atraumatic and normocephalic head. The neck was soft and supple, and the chest was clear to auscultation bilaterally without tenderness. Tachycardia with S1 and S2 sound on auscultation without any murmurs or rubs, soft and non-tender abdomen, and cranial nerves II-XII grossly intact without focal neurological deficits were observed. Integumentary examination revealed an erythematous oval rash in the right scapular and sub-scapular region with central clearing, measuring approximately 5 x 3 inches, as shown in Figure 1 and Figure 2.

**Figure 3:**
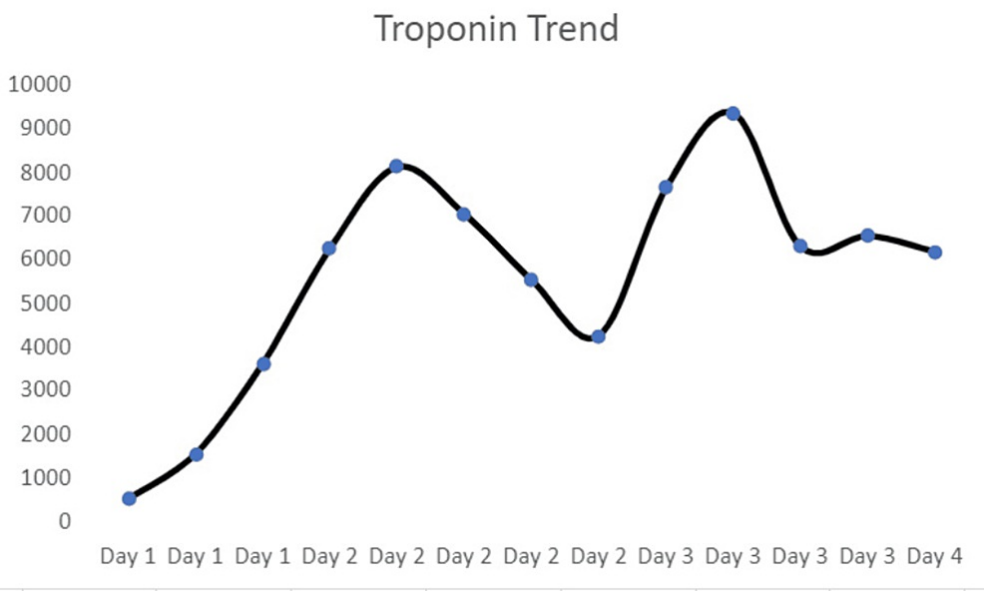
Troponin trend during four days of hospitalization X-axis: days; Y-axis: troponin I in ng/L (normal: <76 ng/L)

The patient's pertinent laboratory values are shown in Table 1. The Troponin trend is depicted in Figure 3.

**Table 1:** Laboratory values

Component	Patient value (normal range)
Sodium	137 (135-145 mEq/L)
Potassium	4.3 (3.5-5.0 mEq/L)
Creatinine (Cr)	0.87 (0.7-1.3 mg/dL)
Thyroid-stimulating hormone (TSH)	0.9 (0.4-4.5 uIU/ml)
White blood cells (WBCs)	14.5 (4000-11000 x 10^3^ cells/ul)
Hemoglobin (Hgb)	16.4 (13.5-17.5 g/dL)
Platelets	237 (150-450 10^3^/uL)
Alanine transaminase (ALT)	31 (7-56 U/L)
Aspartate transaminase (AST)	31 (8-33 U/L)

**Figure 4:**
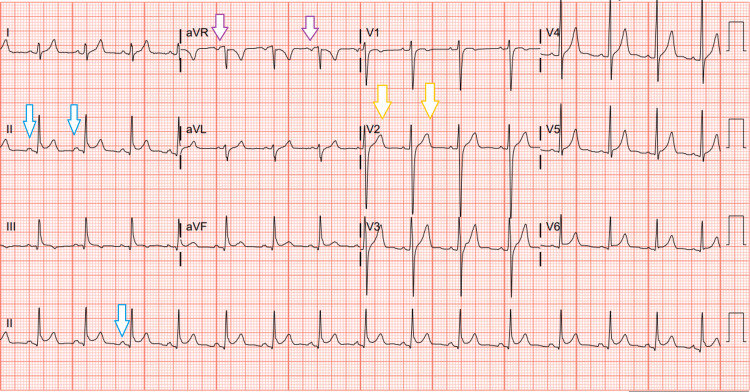
EKG of the patient EKG shows ST-segment elevation in V2 (yellow arrows), PR depressions in II (blue arrows), and PR elevation in AVR (purple arrows), consistent with pericarditis

EKG was significant for ST-segment elevations, as depicted in Figure 4.

The urine drug screen (UDS) was positive for cannabinoids, as he used medical marijuana for OCD. Chest X-ray was negative for any acute pathology. As pulmonary embolism was one of the differentials based on chest pain and tachycardia, a CT angiogram of the chest was obtained, which ruled out pulmonary embolism. The patient was given aspirin 324 mg and colchicine 0.6 mg and admitted with a presumptive diagnosis of acute pericarditis due to his EKG changes and positional chest pain. An echocardiogram revealed more than 70% ejection fraction without segmental wall motion abnormality or pericardial effusion. Although the Lyme antibody was negative, the patient was diagnosed with Lyme-induced pericarditis, as initial Lyme antibody testing can be negative and should be repeated within a few weeks. The patient was started on ibuprofen 400 mg Q8H, colchicine 0.6 mg BID, and intravenous doxycycline. During hospitalization, telemetry leads were significant for episodes of non-sustained ventricular tachycardia intermittently. Daily EKG showed no new ischemic changes but continued to demonstrate ST changes along with PR depression.

After a few days of hospitalization, the patient’s palpitation and shortness of breath resolved while his chest pain gradually improved. Physical examination revealed the evolution of the central clearing of the rash as shown above. Troponin peaked at 9382 ng/L and then started downtrending. Doxycycline was switched to intravenous ceftriaxone. The patient also had one episode of diarrhea, and the colchicine dose was reduced to 0.3 mg BID due to its known gastrointestinal adverse effects. The patient was discharged on oral doxycycline for 14 days and colchicine for six weeks. He was advised to undergo repeat Lyme testing (as the initial results can be negative, and in such cases, repeat testing within a few weeks is recommended) and follow up with outpatient cardiology for further evaluation. The patient did not follow up with the primary provider and was eventually lost to follow-up.

## Discussion

The bacterial agent responsible for Lyme disease is a spirochete called *Borrelia burgdorferi (B. burgdorferi)*, consisting of approximately 20 genospecies. *B. burgdorferi* spreads through the bite of tick species belonging to the genus *Ixodes*. In North America, the *Ixodes* species that transmit Lyme disease is predominantly *Ixodes scapularis* [1]. *Ixodes* ticks follow a metamorphic life-cycle pattern. By feeding on affected zoonotic species (most commonly deer and other small animals), larval ticks pick up spirochetes. The spirochetes are transferred via tick-bites to humans during outdoor activities, especially in the summer [3-4].

Once the bacterium inoculates in the dermis, it undergoes significant changes in gene expression to adapt to the increased temperature, altered pH, and differences in nutrient availability. The bacterium undergoes antigenic variation of surface lipoproteins to evade the host immune response [5]. It replicates and migrates along the plane of the skin and towards the dermal microvasculature. It also releases pathogen-associated molecular patterns, which attract the dendritic cells, macrophages, and circulating immune cells. The ensuing inflammatory response gives rise to the hallmark skin lesion, erythema migrans. Once the bacterium gains access to the vasculature, it disseminates by blood and, via transmigration, invades target organs, primarily the joints, heart, and nervous system [3]. Inflammation generated by host immune responses contributes to pathogenesis and disease symptoms [5].

Early localized, early disseminated, and late disseminated are the three stages of the disease. The red ring-like spreading rash of erythema migrans at the location of a recent tick bite indicates the early localized illness. The rash takes several days or weeks to spread. The early disseminated infection manifests as erythema migrans and neurological and cardiac symptoms spreading over weeks to months. The most frequent neurological manifestation is motor or sensory radiculoneuritis, which manifests as neuropathic pain and headaches; and cranial neuritis, which typically affects the seventh cranial nerve and can involve meninges [5].

Lyme carditis is an uncommon complication of Lyme, with recent studies reporting an incidence of 1% in the United States and 0.3-4% in Europe. Although there is no significant gender difference in the prevalence of Lyme disease, Lyme carditis more commonly affects men, with a male-to-female ratio of 3:1 [4]. This is consistent with the demographic profile of our patients. Lyme carditis is believed to occur due to direct myocardial invasion by *B. burgdorferi* with a resultant exaggerated inflammatory response that is predominantly macrophagic and lymphocytic. Furthermore, there is evidence of cross-reactivity between anti-*Borrelia* antibodies and cardiac tissues, which suggests that autoimmunity may contribute to the exaggerated inflammatory response [4,6].

The symptomatic presentation of Lyme carditis is variable, with some patients remaining asymptomatic and unaware of any underlying cardiac dysfunction. In contrast, others experience light-headedness, syncope, dyspnea, palpitations, or chest pain [7]. Most cases of Lyme myopericarditis are asymptomatic. However, there have been reports of patients experiencing symptoms that mimic acute coronary syndrome. These patients presented with chest pain, EKG findings significant for ST-segment depression/elevation, PR depression/elevation or T wave inversions, and elevated cardiac biomarkers [6]. Our patient presented with complaints of chest pain and shortness of breath, EKG findings significant for ST-segment elevation, PR depression and elevation, and elevated troponins.

In cases of suspected Lyme carditis, monitoring cardiac activity and function with telemetry and imaging is vital. EKG can suggest conduction system impairment. An echocardiogram can provide information about myopericardial involvement, which may manifest as pericardial effusion or depressed systolic function with diffuse hypokinesia or cardiomegaly, but a definitive diagnosis requires either cardiac MRI or cardiac biopsy [4-6]. Cardiac MRI helps to confirm the diagnosis and establish a prognosis. It may reveal inflammation in the myocardium triggered by spirochetes or pericardial signal enhancement, indicating pericarditis-induced irritation [4].

An accurate diagnosis of Lyme myopericarditis requires relevant history, clinical findings, and laboratory data. Serologic tests are the gold standard for diagnosing Lyme disease [8]. These tests can be grouped into direct methods, which detect the spirochete by culture or polymerase chain reaction (PCR), and indirect methods, such as ELISA and western blot, which detect antibodies. Direct tests lack sensitivity for Lyme disease; hence, indirect testing is preferred. A standard two-tier testing strategy is recommended, with an initial enzyme immunoassay and then a western blot assay for specimens yielding positive or equivocal results. However, the Infectious Diseases Society of America is revising its current guidelines for the standard two-tiered approach. The revised strategy is called the two-step ELISA algorithm. It includes testing with early-generation ELISA using whole cell extracts and the newer generation ELISA using antibodies targeting recombinant proteins VisE C6 peptide. This approach has shown higher sensitivity than standard two-tiered testing [9]. ELISA testing does have some limitations in detecting early infection because of the limited antigen expression at this stage. False-negative results are not uncommon in the early phase. If the diagnosis is clinically suspected, the ELISA test may be repeated within two to six weeks to look for seroconversion, and the patient should be empirically treated for Lyme carditis [8].

Lyme pericarditis has a favorable prognosis. In patients with symptoms suggestive of Lyme pericarditis, empiric treatment should be administered immediately, even before Lyme serology is processed. Based on data from case reports and expert opinion, the optimal antibiotic regimen and route is IV ceftriaxone 2 g once daily in adults and 50-75 mg/kg once daily in children. IV ceftriaxone is considered the first-line therapy; other appropriate alternatives include IV cefotaxime (2 g IV twice daily) or high-dose penicillin G. Serologically confirmed Lyme disease should be treated intravenously followed by oral antibiotics for 14-21 days [7]. If substantial improvement enables a patient to be discharged before day 14, a two-week course of oral antibiotics is sufficient [5]. Appropriate oral antibiotics include doxycycline, 100 mg orally twice daily; amoxicillin, 500 mg orally three or four times daily; and cefuroxime, 500 mg twice daily [7].

## Conclusions

Lyme disease has a variable presentation, and its course is usually benign. However, cardiovascular involvement can typically lead to fatal outcomes, including third-degree heart block, which might need a permanent pacemaker placement. A negative test in the initial phase of the disease cannot rule out the disease. An initial negative test should be followed by a repeat test in a few weeks according to the guidelines, and a negative test should not delay the treatment, the absence of which can lead to long-term complications.
